# LRP1 in vascular mural cells modulates cerebrovascular integrity and function in the presence of *APOE4*

**DOI:** 10.1172/jci.insight.163822

**Published:** 2023-04-10

**Authors:** Hiroshi Oue, Yu Yamazaki, Wenhui Qiao, Chen Yuanxin, Yingxue Ren, Aishe Kurti, Francis Shue, Tammee M. Parsons, Ralph B. Perkerson, Keiji Kawatani, Ni Wang, Skylar C. Starling, Bhaskar Roy, Ioana-Emilia Mosneag, Tomonori Aikawa, Marie-Louise Holm, Chia-Chen Liu, Yasuteru Inoue, Patrick M. Sullivan, Yan W. Asmann, Betty Y.S. Kim, Guojun Bu, Takahisa Kanekiyo

**Affiliations:** 1 Department of Neuroscience,; 2 Department of Quantitative Health Sciences, and; 3 Center for Regenerative Medicine, Mayo Clinic, Jacksonville, Florida, USA.; 4 Department of Medicine, Duke University School of Medicine, Durham, North Carolina, USA.; 5 Department of Neurosurgery, The University of Texas MD Anderson Cancer Center, Houston, Texas, USA.

**Keywords:** Neuroscience, Alzheimer disease, Dementia, Mouse models

## Abstract

Cerebrovasculature is critical in maintaining brain homeostasis; its dysregulation often leads to vascular cognitive impairment and dementia (VCID) during aging. VCID is the second most prevalent cause of dementia in the elderly, after Alzheimer’s disease (AD), with frequent cooccurrence of VCID and AD. While multiple factors are involved in the pathogenesis of AD and VCID, *APOE4* increases the risk for both diseases. A major apolipoprotein E (apoE) receptor, the low-density lipoprotein receptor-related protein 1 (LRP1), is abundantly expressed in vascular mural cells (pericytes and smooth muscle cells). Here, we investigated how deficiency of vascular mural cell LRP1 affects the cerebrovascular system and cognitive performance using vascular mural cell–specific *Lrp1*-KO mice (*smLrp1^–/–^*) in a human *APOE3* or *APOE4* background. We found that spatial memory was impaired in the 13- to 16-month-old APOE4 *smLrp1^–/–^* mice but not in the APOE3 *smLrp1^–/–^* mice, compared with their respective littermate control mice. These disruptions in the APOE4 *smLrp1^–/–^* mice were accompanied with excess paravascular glial activation and reduced cerebrovascular collagen IV. In addition, blood-brain barrier (BBB) integrity was disrupted in the APOE4 *smLrp1^–/–^* mice. Together, our results suggest that vascular mural cell LRP1 modulates cerebrovasculature integrity and function in an *APOE* genotype–dependent manner.

## Introduction

Alzheimer’s disease (AD) is the most common form of dementia in the elderly (1–3). Approximately 75% of AD autopsy cases possess some extent of cerebrovascular pathologies: macroinfarcts, microinfarcts, atherosclerosis, arteriolosclerosis, or cerebral amyloid angiopathy (4). Vascular cognitive impairment and dementia (VCID) is caused by a spectrum of vascular brain pathologies, frequently coinciding with AD (5–8). While amyloid-β (Aβ), phosphorylated tau accumulation and progressive neurodegeneration are hallmarks of AD, there is accumulating evidence that associates cerebrovascular dysregulation with AD development (9, 10). Thus, it is critical to define the molecular cerebrovascular contributions to dementia pathogenesis.

Cerebrovasculature is composed of endothelial cells, vascular mural cells (i.e., vascular smooth muscle cells [SMCs] and pericytes), and extracellular matrix basement membrane layers, surrounded by astrocytic end-feet (9, 11). Vascular mural cells (VMCs) play a critical role in regulating cerebral blood flow and blood-brain barrier (BBB) integrity. While apolipoprotein E (apoE) mediates lipid metabolism in the brain, apoE also impacts AD pathogenesis and neurovascular function through Aβ-independent mechanisms (12, 13). *APOE4* is associated with reduced cerebral blood flow in normal-aging individuals (14), exacerbated white matter hyperintensities (15), and BBB dysfunction (16) when compared with *APOE3*. Consistently, evidence shows that *APOE4* disturbs cerebrovascular homeostasis maintenance ability (17–19). Low-density lipoprotein receptor-related protein 1 (LRP1) is a major apoE receptor and is abundantly expressed in several different brain cell types: neurons, glia, and vascular SMCs. LRP1 is an efficient endocytic receptor regulating a variety of cellular properties involved in pathophysiological conditions (20, 21). Hence, we hypothesize that LRP1 is involved in multiple dementia-related pathogenic pathways (22). Our previous report demonstrated that conditional deletion of VMC-LRP1 exacerbates Aβ deposition in APP/PS1 mice (23). However, our knowledge is limited regarding how *APOE4* and VMC-LRP1 contribute to VCID-related phenotypes independently of Aβ pathology. Therefore, we generated VMC-specific LRP1-KO (*smLrp1^–/–^*) mice expressing human *APOE3* or *APOE4*. Here, we show that deletion of VMC-LRP1 has *APOE* genotype–dependent effects on cerebrovascular modulation and cognitive performance.

## Results

### Altered neurobehaviors in smLrp1^–/–^ mice with APOE4.

To investigate the isoform-dependent role of VMC-LRP1 homeostatic regulation, we generated *smLrp1^–/–^* mice by crossing *Lrp1^fl/fl^* and *sm22α-Cre^+/–^* mice (23), followed by breeding these mice with either APOE3–targeted replacement (APOE3-TR) or APOE4-TR mice (24). Brain sections from 13- to 16-month-old APOE3 control, APOE3 *smLrp1^–/–^*, APOE4 control, and APOE4 *smLrp1^–/–^* mice were stained for LRP1 and a VMC marker α-smooth muscle actin (αSMA), validating LRP1 depletion in penetrating arteries in both APOE3 *smLrp1^–/–^* and APOE4 *smLrp1^–/–^* mice (Supplemental Figure 1A; supplemental material available online with this article; https://doi.org/10.1172/jci.insight.163822DS1). Evident atherosclerosis was not detected in aorta of the mice (Supplemental Figure 1B). We also did not observe significant effects of VMC-LRP1 on cortical apoE levels or total cholesterol and triglyceride levels in the plasma (Supplemental Figure 1, C–F).

**Figure 1:**
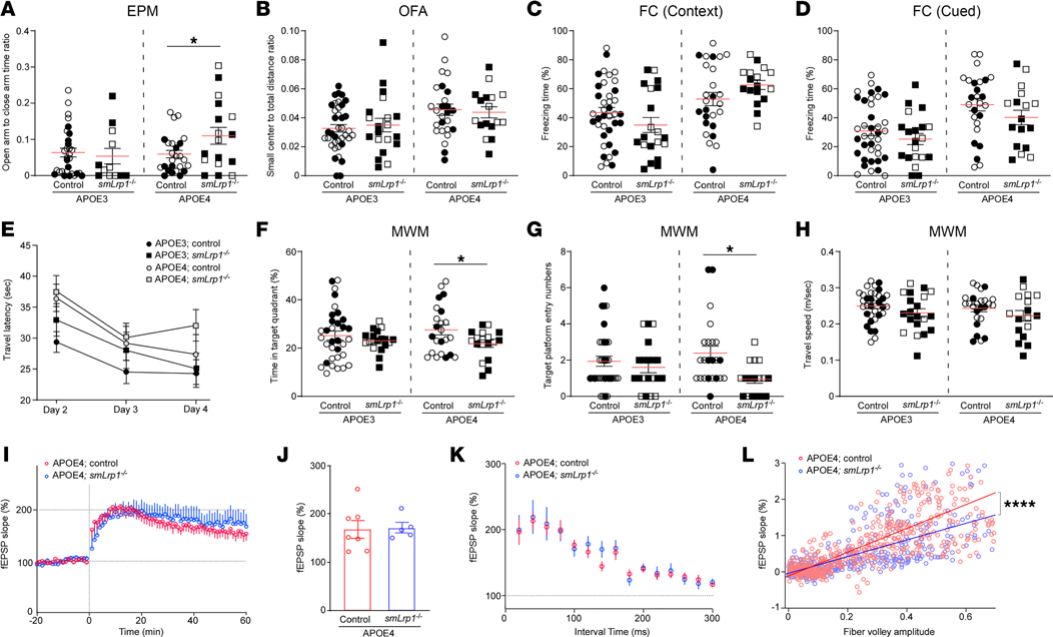
LRP1 deletion in vascular mural cells impairs spatial memory in the mice with *APOE4*. (**A**–**H**) APOE3 control (male; *n* = 14, female; *n* = 20), APOE3 *smLrp1*^–/–^ (male; *n* = 13, female; *n* = 7), APOE4 control (male; *n* = 10, female; *n* = 16), and APOE4 *smLrp1*^–/–^ mice (male; *n* = 9, female; *n* = 8) were subjected to neurobehavior analyses at the age of 13–16 months. The ratio of the time spent in open arms to close arms in the elevated plus maze (EPM) test (**A**), the small center to total distance ratio (**B**) in the open filed assay (OFA), and the percentage of time with freezing behavior in the contextual (**C**) and cued (**D**) fear conditioning (FC) tests are shown. Travel latency to invisible platform in the Morris water maze (MWM) test plotted against the training days (**E**), the percentage of time in target quadrant (**F**), the entry numbers in target quadrant (**G**), and travel speed (**H**) are shown. Closed circles/squares and open circles/squares indicate male and female mice, respectively. Data are shown as mean ± SEM. *P < 0.05 by Student’s *t* test between control and *smLrp1*^–/–^ mice in each *APOE* genotype. (**I**–**L**) Normalized fEPSP responses to field stimulation in the CA1 region of hippocampal slices (14–29 slices from 5–7 mice/group) (**I**) from 13- to 16-month-old APOE4 control and APOE4 *smLrp1*^–/–^ mice are plotted. Values after 60 minutes of stimulation are expressed as the mean ± SEM (**J**). Plots of fEPSP slope versus interval time (**K**) and fiber volley amplitude (**L**) are shown. ****P < 0.0001 by repeated-measures 1-way ANOVA.

To determine the effects of VMC-LRP1 deletion on behavioral performance, the 13- to 16-month-old mice were subject to a behavioral battery: elevated plus maze (EPM) test, open-field analysis (OFA), contextual and cued fear conditioning (CFC) tests, and the Morris water maze (MWM) test. The EPM detected increased time spent in the open arm as opposed to the closed arm in APOE4 *smLrp1^–/–^* mice compared with APOE4 control mice (Figure 1A). No differences were observed in the ratio of traveled distance in the center of the field to total distance in the OFA (Figure 1B). This result indicates that VMC-LRP1 deletion may cause disinhibition-like behavior rather than anxiety in the mice with *APOE4*. FC found no significant differences in context-associated memory (Figure 1C) or auditory cue–associated memory (Figure 1D) between APOE4 *smLrp1^–/–^* and APOE4 control mice. Although MWM platform latency during training did not differ between groups (Figure 1E), we found that APOE4 *smLrp1^–/–^* mice spent less time and had fewer entries in the target quadrant (Figure 1, F and G) than APOE4 control mice during probe trials. Travel speed was unaffected (Figure 1H). MWM results indicate impaired memory preservation in APOE4 *smLrp1^–/–^* mice. APOE3 *smLrp1^–/–^* mice did not show any changes in the behavior tests when compared with APOE3 control mice (Figure 1, A–H), implying that VMC-LRP1 deletion induces isoform-dependent abnormal behaviors and disturbed spatial memory.

Theta-burst stimulation–induced (TBS-induced) long-term potentiation (LTP) in hippocampal CA1 showed no obvious differences in normalized field excitatory postsynaptic potentials (fEPSP) (Figure 1, I and J) or presynaptic function (Figure 1K) between APOE4 *smLrp1^–/–^* and APOE4 control mice. However, we found that fiber volley amplitude increase led to modest fEPSP slope change in APOE4 *smLrp1^–/–^* mice compared with APOE4 control mice (Figure 1L), indicating basic synaptic transmission reduction.

### Modified brain transcriptome profiles in smLrp1^–/–^ mice with APOE4.

**Figure 2:**
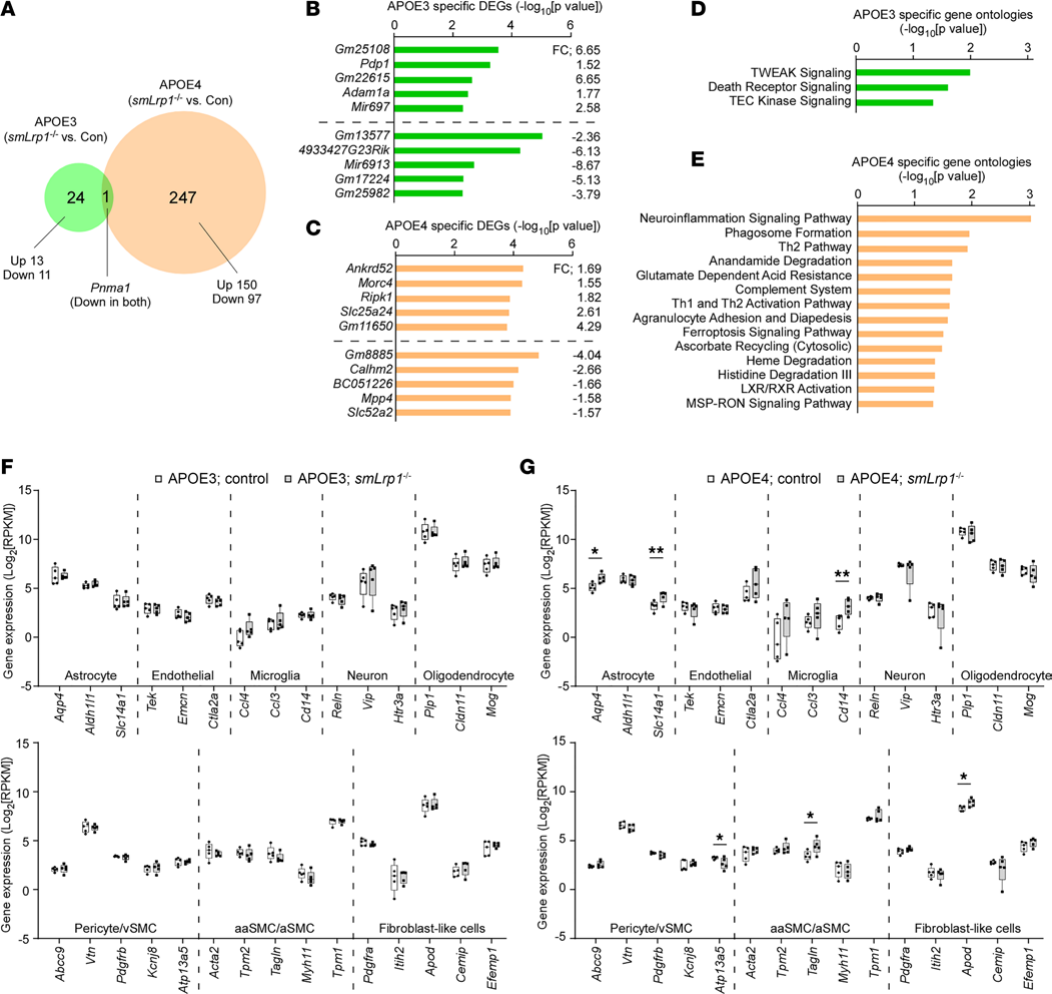
LRP1 deletion in vascular mural cells exacerbates neuroinflammation in the mice with *APOE4*. Transcriptomes in the cortical samples from 13- to 16-month-old male APOE3 control, APOE3 *smLrp1*^–/–^, APOE4 control, and APOE4 *smLrp1*^–/–^ mice were assessed through RNA-Seq (*n* = 5/group). (**A**) Venn diagram of the DEGs affected by LRP1 deletion in vascular mural cells in the mice with *APOE3* and *APOE4* background (*P* < 0.01, |fold change| > 1.5) is shown. (**B**–**E**) Top 10 DEGs in the mice with *APOE3* (**B**) and *APOE4* (**C**) background, and GO terms changed with *P* < 0.05 in the mice with *APOE3* (**D**) and *APOE4* (**E**) background are shown. (**F** and **G**) Effects of LRP1 deletion in vascular mural cells on the transcriptome expression of selected marker genes for major brain cell types (astrocyte, endothelial cell, microglia, neuron, and oligodendrocyte) as well as vascular mural cell types, including pericyte/venous smooth muscle cell (vSMC), arterial SMC (aSMC)/arteriole SMC (aaSMC), and fibroblast-like cells through the RNA-Seq data in the mice with *APOE3* (**F**) and *APOE4* (**G**) background are shown. Horizontal lines, boxes, and whiskers correspond to median, interquartile range (IQR), and minimum/maximum, respectively. *P < 0.05; **P < 0.01.

We conducted bulk RNA-Seq with cortical samples from 13- to 16-month-old APOE3 control, APOE3 *smLrp1^–/–^*, APOE4 control, and APOE4 *smLrp1^–/–^* mice. We assessed VMC-LRP1 deletion induced differentially expressed genes (DEGs) (*P* < 0.01, |fold change| > 1.5) and found 25 and 248 gene changes in the mice with *APOE3* and *APOE4* backgrounds, respectively. Only *Pnma1* overlapped between genotype backgrounds and was downregulated in both groups (Figure 2, A–C). Pathway analysis showed that “*TNF-related weak inducer of apoptosis (TWEAK) signaling*”, “*Death receptor signaling*,” and “*TEC tyrosine-protein kinase signaling*” were identified as top-ranked pathways in *APOE3-*specific ontologies (Figure 2D). The “*Neuroinflammation signaling pathway*” was the top-ranked *APOE4*-related pathway (Figure 2E). We focused transcriptome analysis on astrocytes, endothelial cells, microglia, neurons, oligodendrocytes (25), and vascular-related cell types (pericyte/venous SMC, arterial SMC/arteriole SMC, and fibroblast-like cells) (26). We found higher expression of astrocyte markers *Aqp4* and *Slc14a1*, a microglia marker *Cd14*, a SMC marker *Tagln*, and a fibroblast-like cell marker *Apod* — but lower expression of the pericyte marker *Atp13a5 —* in APOE4 *smLrp1^–/–^* mice compared with APOE4 control mice. No differences were detected in *APOE3* animals (Figure 2, F and G).

### Astrogliosis in smLrp1^–/–^ mice with APOE4.

**Figure 3:**
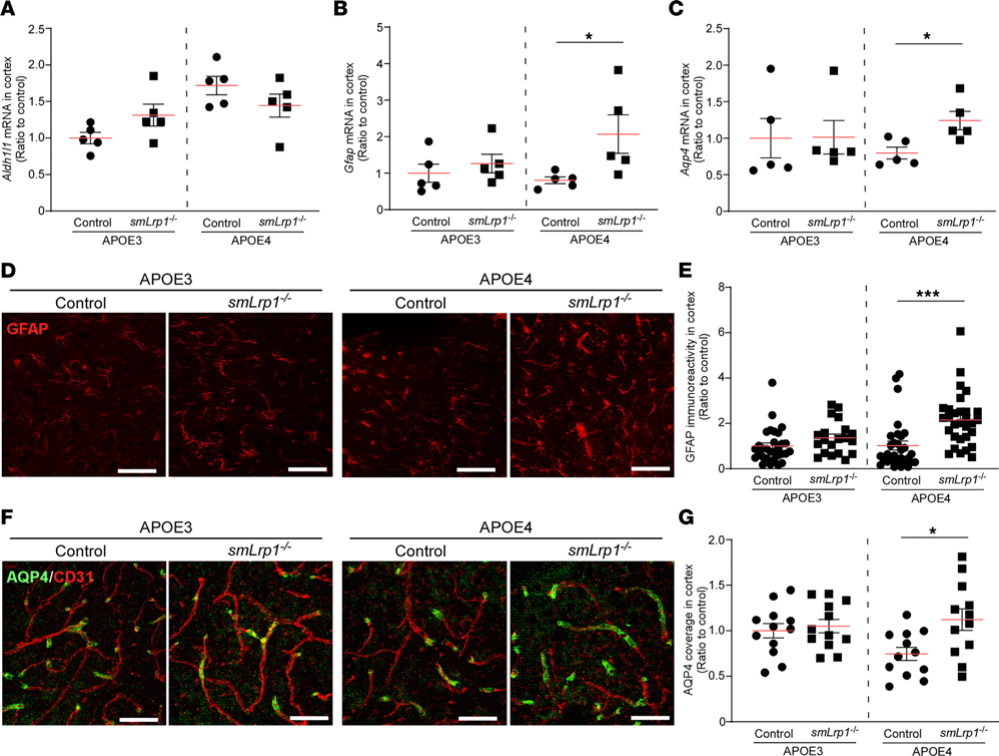
LRP1 deletion in vascular mural cells causes astrogliosis in the mice with *APOE4*. (**A**–**C**) The mRNA expression of *Aldh1l1* (**A**), *Gfap* (**B**), and *Aqp4* (**C**) was measured by qPCR in the cortical samples from male 13- to 16-month-old APOE3 control, APOE3 *smLrp1*^–/–^, APOE4 control, and APOE4 *smLrp1*^–/–^ mice (*n* = 4–5/group). Each mRNA expression was normalized to *Hprt* mRNA expression and shown as a ratio to that of APOE3 control. (**D**) GFAP was stained in frozen brain sections from 13- to 16-month-old male APOE3 control, APOE3 *smLrp1*^–/–^, APOE4 control, and APOE4 *smLrp1*^–/–^ mice. Scale bars: 100 μm. (**E**) Total fluorescence intensity of GFAP in the cortical sections was quantified by ImageJ software (NIH; 20–30 regions from 4 mice/group) and shown as a ratio to that of APOE3 control. (**F**) AQP4 and CD31 were stained in frozen brain sections from 13- to 16-month-old male APOE3 control, APOE3 *smLrp1*^–/–^, APOE4 control, and APOE4 *smLrp1*^–/–^ mice. Scale bars: 50 μm. (**G**) The percentage of coverage against CD31^+^ endothelial by AQP4 in the cortical sections was quantified by ImageJ software (12 regions from 4 mice/group) and shown as a ratio to that of APOE3 control. Data are shown as mean ± SEM. *P < 0.05, ***P < 0.001 by Student’s *t* test between control and *smLrp1*^–/–^ mice in each *APOE* genotype.

Consistent with the bulk RNA-Seq results, quantitative PCR (qPCR) validated mRNA upregulation of activated astrocyte markers *Gfap* and *Aqp4*, but not a pan-astrocyte marker *Aldh1l1*, in APOE4 *smLrp1^–/–^* mice (Figure 3, A–C). Immunostaining validated that VMC-LRP1 deletion increased GFAP immunoreactivity in the mice with *APOE4* but not those with *APOE3* (Figure 3, D and E). There was also increased AQP4^+^ astrocytic end-feet coverage of brain capillaries in APOE4 *smLrp1^–/–^* mice (Figure 3, F and G). These results indicate that VMC-LRP1 modulates cerebrovascular astrogliosis in the presence of *APOE4*.

We focused on homeostatic microglia, disease-associated microglia (DAM), and activated response microglia (ARM) genes (27–29) in our RNA-Seq data set. We found higher expressions of *Hexb*, *Axl*, *Ccl6*, *Lilrb4*, *Cst7*, *Spp1*, and *Hif1a* in APOE4 *smLrp1^–/–^* mice than APOE4 control mice and no differences in those with *APOE3* (Supplemental Figure 2A). Expression of homeostatic microglia gene *Cx3cr1*, major stage 1 DAM gene *Tyrobp*, and major stage 2 DAM gene *Trem2* was unaffected by VMC-LRP1 deletion in 13- to 16-month-old APOE4 mouse cortices. In contrast, the ARM gene *Spp1* was increased in APOE4 *smLrp1^–/–^* mice (Supplemental Figure 2, B–E). Western blotting did not find evident microglia marker Iba-1 differences in cortices among 13- to 16-month-old mice (Supplemental Figure 2F). These results suggest that VMC-LRP1 deletion predominantly activates astrocytes as opposed to microglia in the mice with *APOE4*. *Spp1* is also abundantly expressed in fibroblast-like cells; thus, perivascular fibrosis may be associated with APOE4 *smLrp1^–/–^* astrogliosis.

### Reduced cerebrovascular collagen IV in smLrp1^–/–^mice with APOE4.

**Figure 4:**
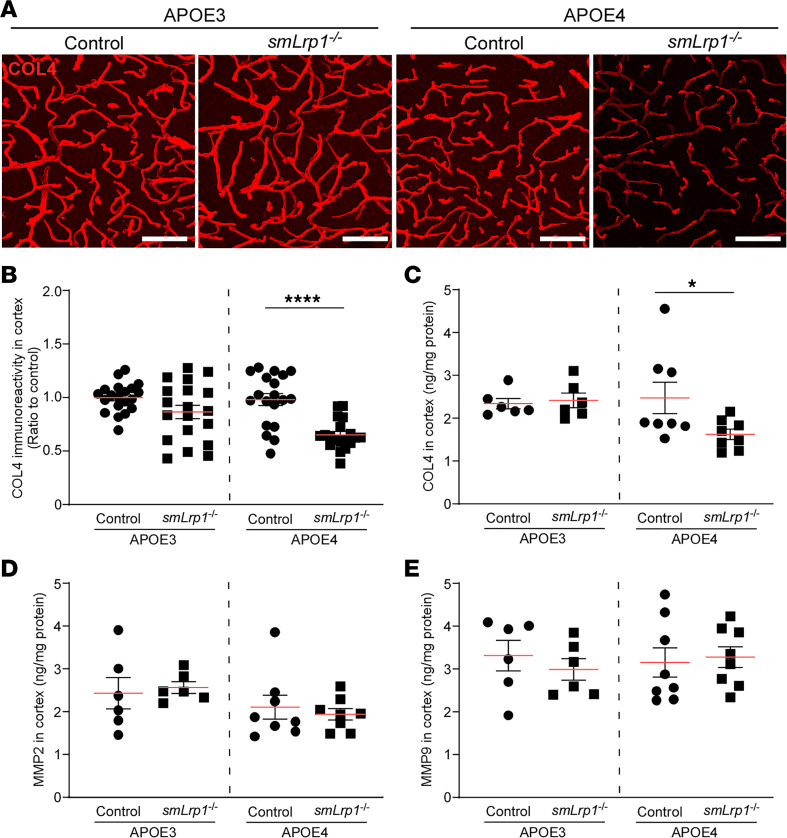
LRP1 deletion in vascular mural cells decreases collagen-IV along brain capillaries in the mice with *APOE4*. (**A** and **B**) Collagen IV was stained in frozen brain sections from 13- to 16-month-old male APOE3 control, APOE3 *smLrp1*^–/–^, APOE4 control, and APOE4 *smLrp1*^–/–^ mice. Scale bars: 100 μm. Total fluorescence intensity of collagen IV in the cortical sections was quantified by ImageJ software (19–20 regions from 4 mice/group) and shown as a ratio to that of APOE3 control. (**C**–**E**) The levels of collagen IV (**C**), MMP2 (**D**), and MMP9 (**D**) in the cortex from 13- to 16-month-old male APOE3 control, APOE3 *smLrp1*^–/–^, APOE4 control, and APOE4 *smLrp1*^–/–^ mice (*n* = 6–8/group) were determined by ELISA. Data are shown as mean ± SEM. *P < 0.05, ****P < 0.0001 by Student’s *t* test between control and *smLrp1*^–/–^ mice in each *APOE* genotype.

We further explored the molecular networks affected by VMC-LRP1 deletion by conducting weighted gene coexpression network analysis (WGCNA) on the bulk RNA-Seq data set. We identified 1 upregulated (dark turquoise) and 1 downregulated (dark green) module in APOE4 *smLrp1^–/–^* compared with APOE4 control mice (Supplemental Figure 3). “*Cyclohydrolase activity*” was identified as a top-ranked pathway in the dark green module. This pathway was enriched with *Pcdhg* cluster genes, which correlated with plasma membrane component and cell adhesion pathways (Supplemental Figure 3). *Pcdhg* genes are expressed in cerebrovascular endothelial cells, where *Pcdhgc3* KO has been shown to modulate tight junction protein levels in vitro (30). Brain capillary tight junction (OCLN, CLDN5 and ZO1) and pericyte coverage remained consistent between APOE4 *smLrp1^–/–^* mice and control mice (Supplemental Figure 4). In contrast, there were significant reductions of vascular collagen IV in the cortex of 13- to 16-month-old APOE4 *smLrp1^–/–^* mice compared with APOE4 control mice but not those with *APOE3* (Figure 4, A and B). Consistently, validation through ELISA also showed lower cortical collagen IV levels in APOE4 *smLrp1^–/–^* mice than APOE4 control mice (Figure 4C), suggesting that VMC-LRP1 deletion compromises the cerebrovascular basement membrane components in an isoform-dependent manner. MMP2 (Figure 4D) and MMP9 (Figure 4E) were not affected in the cortex of APOE4 *smLrp1^–/–^* mice, while they are major LRP1 ligands associated with collagen IV degradation (17).

### Disrupted BBB integrity in smLrp1^–/–^ mice with APOE4.

**Figure 5:**
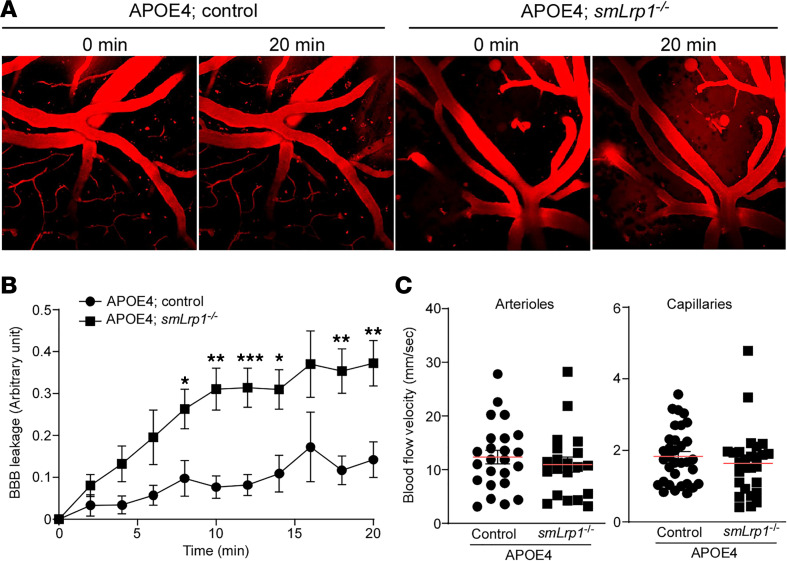
LRP1 deletion in vascular mural cells disturbs BBB integrity in the mice with *APOE4*. (**A**) Dextran (40 kDa) were i.v. injected into APOE4 control and APOE4 *smLrp1*^–/–^ mice at the age of 13–16 months. (**B**) The leakage of dextran into cortical parenchyma was continuously monitored by 2-photon microscopy in different regions (11 regions from 3–4 mice/group) from APOE4 control and APOE4 *smLrp1*^–/–^ mice. (**C**) Blood flow velocity was measured in different arterioles (20–25 vessels) and capillaries (26–31 vessels) from APOE4 control (*n* = 3/group) and APOE4 *smLrp1*^–/–^ mice (*n* = 4/group). Data are shown as mean ± SEM. *P < 0.05, **P < 0.01, and ***P < 0.001 by Student’s *t* test between control and *smLrp1*^–/–^ mice in each *APOE* genotype.

Next, we investigated endothelial cell barrier integrity in the cortex from 13- to 16-month-old APOE3 control, APOE3 *smLrp1^–/–^*, APOE4 control, and APOE4 *smLrp1^–/–^* mice**,** as collagen IV plays a critical role in the cerebrovascular system. While endogenous plasma protein detection in the brain parenchyma represents BBB leakage, we did not detect significant differences in the leakage of albumin or brain levels of IgG and fibrinogen among mouse groups (Supplemental Figure 5). Conversely, i.v. injected fluorescently labeled dextran showed a time-dependent increase of cortical parenchyma leakage in APOE4 *smLrp1^–/–^* mice compared with control mice, which was visualized through 2-photon in vivo imaging (Figure 5, A and B). There were no differences in arterial or capillary cerebral blood flow velocity between APOE4 control and APOE4 *smLrp1^–/–^* mice (Figure 5C). These results suggest that *APOE4* and VMC-LRP1 deletion may specifically lead to the leakage of small molecules from blood flow by disturbing BBB integrity.

## Discussion

VMC-LRP1 plays a pivotal role in vascular wall integrity and contractility by modulating multiple pathways (31): PDGF signaling (32, 33), CTGF and HtrA1 expression (34), and calcium influx (35). VMC-LRP1 deletion has also been reported to compromise Aβ clearance (23, 36) and impair BBB function (17). LRP1 mediates uptake of a variety of ligands including apoE, α2-macroglobulin, and tissue plasminogen activator (20, 37). We demonstrated that VMC-specific LRP1 KO leads to VCID-related phenotypes such as cognitive decline and BBB dysregulation in middle-aged mice with *APOE4*, while the deletion did not induce evident phenotypes in *APOE3* carriers. Therefore, *APOE4*-carrying mice might be more vulnerable to VMC-LRP1 deletion, suggesting isoform-dependent mechanisms mediating cerebrovascular function, though further studies are required.

VMCs not only regulate vascular reactivity and tone (38) but are also pivotal in collagen dynamic regulatory pathways involved in extracellular matrix vessel maintenance and remodeling (39–43). We found cerebrovascular collagen IV reductions and resultant impaired BBB integrity in APOE4 *smLrp1^–/–^* mice. *APOE4* is likely associated with a thinner cerebrovascular basement membrane in patients with AD (44), which can potentially be attributed to its inability to regulate matrix remodeling. Thus, VMC-LRP1 and *APOE4* may synergically exacerbate cerebrovascular basement membrane component reduction. Pericytes and vascular endothelial cell cocultures facilitate mRNA expression of extracellular matrix components in endothelial cells with weaker effects in *APOE4* than *APOE3* pericytes (19). VCM-LRP1–deficient effects on vascular collagen IV may become evident in the presence of *APOE4*. Indeed, *APOE4* is associated with the increased activity of cyclophilin A–MMP9 pathway in pericytes (16, 45). Although further studies are necessary to investigate if LRP1 in pericytes directly mediates the apoE isoform effect, LRP1 deficiency induces the upregulation of cyclophilin A in pericytes (17). Collagen IV is essential for maintaining cerebrovascular function (46), where the deficiency is associated with cerebrovascular microhemorrhages resembling small vessel disease pathology in mice (47, 48). However, there are also conflicting reports of increased microvessel collagen IV in patients with AD (49, 50). It is possible that nonfunctional collagen IV selectively accumulates on microvessels due to compromised extracellular matrix remodeling during AD development.

APOE4 *smLrp1^–/–^* mice showed astroglial activation and increased AQP4^+^ astrocyte end-feet around brain capillaries. AQP4 is the most abundant water channel in the brain and is increased during brain edema in cerebrovascular diseases (51). While AQP4 facilitates edema clearance following vascular damage, it may aggravate edema formation due to astrocytic oxygen and glucose deprivation, resulting in BBB breakdown (51). Evidence shows that AQP4-transgenic mice have exacerbated brain swelling following acute water intoxication (52). Thus, AQP4 upregulation may be both causatively and consequently involved in BBB dysfunction in APOE4 *smLrp1^–/–^* mice. Interestingly, VMC-apoE4 overexpression leads to astrogliosis in *Apoe*-KO mice (53). VMC-LRP1 deletion should cause paravascular apoE4 accumulation, but no differences were found in cortical apoE4 levels. Thus, astrocytes may be abnormally active around cerebrovasculature after prolonged apoE4 exposure, yielding BBB dysfunction. We found that VMC-LRP1 deletion causes brain immune activation through astrogliosis in *APOE4-*carrying mice. BBB leakage and dysregulation lead to neuroinflammation, where excess brain immune responses disturb endothelial barrier integrity in neurodegenerative diseases (54). Future studies should dissect how apoE isoforms and LRP1 regulate cerebrovascular and glial interaction under physiological and pathological conditions.

In summary, our findings demonstrate that VMC-LRP1 deletion reduces cerebrovascular collagen IV and enhances astrogliosis in paravascular regions, resulting in cognitive impairment in the mice with *APOE4* but not *APOE3*. Although apoE isoforms differentially regulate gliovascular functions, LRP1 may not directly mediate beneficial apoE3 functions or deleterious apoE4 effects in VMCs. We previously found that neuronal LRP1 deletion ameliorates Aβ pathology exacerbated by *APOE4* (55). Therefore, future studies should determine how vascular mural LRP1 deficiency and *APOE4* impacts cerebrovascular functions in the presence of Aβ. Taken together, as LRP1 levels decrease in aging cerebrovasculature (36), our results suggest that LRP1 contributes to VCID pathogenesis in an *APOE* genotype–dependent manner.

## Methods

### Animals.

We generated *smLrp1^–/–^* mice by breeding *Lrp1*-floxed mice with sm22α-driven Cre recombinase transgenic mice (23). S*mLrp1^–/–^* mice were further crossed with APOE3- or APOE4-TR mice (24) to generate APOE3 control (*APOE*^ε3/ε3^; *Lrp1^fl/fl^*; *sm22*α*-Cre^–/–^*), APOE3 *smLrp1^–/–^* (*APOE*^ε3/ε3^; *Lrp1^fl/fl^*; *sm22*α*-Cre^+/–^*), APOE4 control (*APOE*^ε4/ε4^; *Lrp1^fl/fl^*; *sm22*α*-Cre^–/–^*), and APOE4 *smLrp1^–/–^* (*APOE*^ε4/ε4^; *Lrp1^fl/fl^*; *sm22*α*-Cre^+/–^*) mice. Thirteen- to 16-month-old male and female littermates with *APOE3* or *APOE4* were used for experiments.

### Mouse behavioral tests.

All mouse behavioral tests were conducted at the Mayo Clinic Jacksonville Mouse Behavior Core as described previously (53). For the EPM, mice were placed in the center of the elevated maze (50 cm from the floor) consisting of 2 open arms and 2 closed arms with roofless walls (width × length; 10 × 50 cm/each). Movements were monitored and tracked using an overhead camera with ANY-Maze software (Stoelting Co.) for time spent in open and closed arms. For OFA, mice were placed in the center of an open-field arena (width × length × height; 40 × 40 × 30 cm). Their movements were monitored and tracked using an overhead camera for total distance traveled and distance traveled in the center of the open-field arena (width × length; 8 × 8 cm). For CFC, mice were trained with an auditory tone (80 dB for 15 seconds) and electrical foot shock (0.6 mA for 1 seconds) in a chamber (width × length × height; 40 × 40 × 30 cm) with a grid floor and returned to their home cages on day 1. For the context test, freezing behavior was recorded for 5 minutes in an identical testing chamber on day 2. For the cued test, visual and olfactory contextual cues in the environments were modified. Freezing behavior was recorded for 3 minutes with an auditory tone on day 2. Freezing behavior was monitored with an overhead camera using FreezeFrame software (Actimetrics). For the MWM, a transparent platform (10 cm diameter) was placed in the target quadrant in a pool (122 cm diameter) filled with water mixed with nontoxic white paint at 21°C–23°C. For the visible platform test, mice underwent 1 session of 6 pretraining trials where a visible marker was placed on the platform on day 1. When mice did not mount the platform within 60 seconds, they were guided to the platform. For hidden platform training, the platform located in the target quadrant of the pool was submerged 1.5 cm below the surface. Training sessions composed of 6 trials per day were performed on 3 consecutive days (days 2–4). For the probe test, the platform was removed, and mice were placed at the opposite edge of the pool. Behaviors were monitored for 60 seconds on day 5. All behaviors were recorded with a video tracking system for travel latencies, time spent in target quadrant, platform entries, and travel speed.

### Electrophysiological analysis.

fEPSPs were assessed from the mouse hippocampal CA1 stratum radiatum in fresh slices using a glass microelectrode (2–4 mΩ) filled with artificial cerebrospinal fluid composed of: NaCl (125 mM), KCl (2.5 mM), NaH_2_PO_4_ (1.25 mM), NaHCO3 (25 mM), glucose (25 mM), MgCl_2_ (1 mM), and CaCl_2_ (2 mM; Sigma-Aldrich). fEPSPs were evoked through Schaffer collateral stimulation using a 0.1 millisecond biphasic pulse. To measure basic synaptic transmission, stimulation was strengthened incrementally every 0.5–1 mV until the maximum amplitude of the fEPSP was reached. Subsequent stimulation paradigms were induced at the voltage evoking 50%–60% of the maximum fEPSP amplitude in each slice. Baseline fEPSP responses were recorded for 20 minutes, followed by the paired-pulse facilitation (PPF) measurements. PPF recording was performed using a series of 15 paired pulses with an initial 20 millisecond interval and an incremental step of 20 milliseconds. To evoke LTP, a TBS protocol was utilized composed of 5 trains of 4 pulse bursts at 200 Hz, separated by 200 milliseconds, repeated 6 times with an intertrain interval of 10 seconds. Potentiation was measured for 60 minutes as the increase of the mean fEPSP descending slope following TBS normalized to the mean fEPSP descending slope of baseline recordings. All the recordings were analyzed using MATLAB (9.6.0.1072779 R2019a, Mathworks) as described previously (53).

### RNA-Seq.

Total RNAs were extracted from samples using TRIzol RNA Isolation Reagents (Thermo Fisher Scientific) and a RNeasy mini kit with DNase (QIAGEN). RNA integrity number (RIN) of all samples were measured with a 2100 Bioanalyzer (Agilent Technologies) using an RNA 6000 nano kit (Agilent Technologies). RNA samples were sequenced with Illumina HiSeq 4000 at the Mayo Clinic Genome Facility. Reads were mapped to the mouse genome mm10. Raw gene read counts and sequencing quality control were generated using the Mayo Clinic RNA-Seq analytic pipeline, MAP-RSeq Version 3.0 (56). We performed conditional quantile normalization (CQN) on raw gene counts to correct for guanine and cytosine (GC) bias and gene length differences as well as to obtain similar quantile-by-quantile distributions of gene expression levels across samples. Based on the bimodal distribution of the CQN-normalized and log_2_-transformed reads per kb per million (RPKM) gene expression values, genes with average log_2_ RPKM of 0 or more in at least 1 group were considered expressed above detection threshold. Pathway analyses of DEGs were performed using Ingenuity Pathway Analysis (IPA; QIAGEN) (57). Transcriptomics WGCNA was performed using CQN-normalized and log_2_-transformed RPKM values adjusted for animal age. The power of 7 was chosen to build scale-free topology using signed hybrid network (58). Hybrid dynamic tree cutting was utilized based on a minimum module size of 70 genes and a minimum height for merging modules of 0.3. Each module was summarized by module eigengene (ME). Modules were annotated using the WGCNA R function GOenrichmentAnalysis. Genes with high connectivity in the respective modules were considered hub genes. Gene-to-gene connections among top hub genes were visualized using VisANT version 5.51 (59).

### qPCR.

After RNA sample extraction with DNase and a RNeasy mini kit (QIAGEN), reverse transcription was performed using iScript Reverse Transcription Supermix (Bio-Rad). Complementary DNA was added to a reaction mix containing gene-specific primers and SYBR Green Supermix (Bio-Rad). All samples were analyzed with CFX96 Real-Time PCR Detection System (Bio-Rad). The relative gene expression was normalized to *Hprt* expression and assessed using the 2^–ΔΔCT^ method. Primer sequences are as follows *Hprt*, 5′-TCCTCCTCAGACCGCTTTT-3′ (forward [F]) and 5′-CCTGGTTCATCATCGCTAATC-3′ (reverse [R]); *Cx3Cr1*, 5′-CAGCATCGACCGGTACCTT-3′ (F) and 5′-GCTGCACTGTCCGGTTGTT-3′ (R); *Tyrobp*, 5′-GAGTGACACTTTCCCAAGATGC-3′ (F) and 5′-CCTTGACCTCGGGAGACCA-3′ (R); *Trem2*, 5′-GCACCTCCAGGAATCAAGAG-3′ (F) and 5′-GGGTCCAGTGAGGATCTGAA-3′ (R); *Spp1*, 5′-CCATCTCAGAAGCAGAATCTCCTT-3′ (F) and 5′-GGTCATGGCTTTCATTGGAATT-3′ (R); *Aldh1l1*, 5′-CTTCATAGGCGGCGAGTTTGTG-3′ (F) and 5′-CGCCTTGTCAACATCACTCACC-3′ (R); *Gfap*, 5′-TCGAGATCGCCACCTACAG-3′ (F) and 5′-GTCTGTACAGGAATGGTGATGC-3′ (R); and *Aqp4*, 5′-AGCCAGCATGAATCCAGCTCGA-3′ (F) and 5′-TCATAAAGGGCACCTGCCAGCA-3′ (R).

### Biochemical assays.

Brain tissues were homogenized in RIPA buffer (MilliporeSigma) containing complete protease inhibitors (Roche) and PhosSTOP phosphatase inhibitors (Roche). After centrifugation (100,000*g* for 1 hour at 4°C), the supernatant was used for biochemical analysis as described (53). ApoE (53, 55), collagen IV (LS-F20750, LSBio), MMP-2 (MMP200, R&D Systems), and MMP-9 (NBP2-60095, Novus Biologicals) in brain lysate were measured by ELISA. Brain lysate measurements were normalized to total protein concentrations determined by BCA assay (Thermo Fisher Scientific). Total cholesterol and triglycerides in plasma were measured using a Cholesterol Assay Kit (A12216, Thermo Fisher Scientific) and Triglyceride Assay Kit (ab65336, Abcam), respectively. Some brain samples were subjected to Western blotting using anti–Iba-1 antibody (17198, Cell Signaling Technology) and anti–β-actin antibody (3700, Cell Signaling Technology), followed by quantification through LI-COR Odyssey.

### IHC analysis.

IHC analysis was performed as described previously (19, 53, 60). Frozen coronal sections were stained with rabbit-polyclonal anti-LRP1 antibody (homemade) (23), anti-αSMA antibody (A2547, MilliporeSigma), anti-GFAP antibody (MAB360, MilliporeSigma), anti-AQP4 antibody (AB3594, MilliporeSigma), anti–collagen IV antibody (AB756P, MilliporeSigma), anti-albumin antibody (A90-134, Bethyl Laboratories), FITC conjugated anti-CD13 antibody (BDB558744, BD Biosciences), Alexa Fluor 488–conjugated anti–ZO-1 antibody (339188, Invitrogen), Alexa Fluor 488–conjugated anti-OCLN antibody (331588, Invitrogen), or Alexa Fluor 488–conjugated anti-CLDN5 antibody (352588, Invitrogen), followed by incubations with or without Alexa Fluor 488 or Alexa Fluor 568 secondary antibody. In some experiments, sections were subsequently stained with APC-labeled anti-CD31 antibody (BDB561814, BD Biosciences) to visualize endothelial cells. Images were captured by confocal laser-scanning fluorescence microscopy (LSM880, Carl Zeiss). Immunoreactivity was quantified using NIH ImageJ software as described (19, 60). Capillary coverage of AQP4, CD13, ZO-1, OCLN, or CLDN5 were defined as area of colocalization/total CD31^+^ area (19, 60). To assess atherosclerotic lesions in the aorta, frozen sections were subjected to Oil Red O (ab150678, Abcam) and hematoxylin staining (53).

### In vivo 2-photon imaging.

All procedures were adopted from previously published methods with modifications (61). Mice were anesthetized using isoflurane (4% for induction and 1.5%–2% for surgery) and immobilized in a stereotactic apparatus (Model942, Kopf Instruments). Body temperature was monitored and maintained at 37.0°C with a heating blanket (Homeothermic Blanket Systems, Harvard Apparatus). After scalp removal, a 4 mm craniotomy over the primary somatosensory cortex (coordinates: P2, L2) was created using a high-speed microdrill (K1040, Foredom) under a dissecting microscope. A custom-made metal plate and a cover slip (5 mm diameter) were glued onto the skull with dental acrylic cement. Mice were anesthetized using isoflurane (4% for induction and 0.5%–1.0% for imaging) and immobilized on a custom-made microscope stage. An upright laser scanning microscope (BX61WI, Olympus) attached to a Ti:Sapphire pulsed laser system (80 MHz repetition rate, < 100 fs pulse width; Spectra Physics) and software (Prairie view 5.2, Bruker) was used for 2-photon fluorescence imaging. Vasculature was visualized through i.v. Texas Red–conjugated dextran (40 kD, 12.5 mg/kg; Thermo Fisher Scientific) injection. Time-lapse imaging of small cortical subvolumes (20–25 image planes with 1–2 μm axial spacing) was performed for at least 20 minutes to track the permeability in the cerebral cortex, and the interval duration between stack sequence was 2–3 minutes. PMT settings (including gain and offset) and laser excitation power were kept constant during time-lapse imaging. Line scan was performed along the central axis in the single vessel and perpendicular to the single vessel to measure blood flow velocity and vessel diameter, respectively. Arterioles, veins, and capillaries were discriminated by the following 3 criteria: the direction of blood flow from the pial surface, line scan pattern, and vessel diameter. Capillaries were identified by their diameter (<5 μm). Average laser power for imaging was < 50 mW. All images were processed by using the open-source software Fiji (http://fiji.sc/) and a custom-written program with 64-bit Matlab (Version 8.5.0 R2015a, Mathworks). Registration was used to perform intensity-based image alignment at different time points. Matlab-based custom-written script with radon transform algorithm had been used to measure blood flow velocity in the cerebral cortex (62). Maximum intensity projection (MIP) was used to display vasculature morphology for further analysis. To measure the endothelial cell barrier integrity, the mean fluorescence intensity of each region of interest was outlined within the parenchyma and calculated on a minute-by-minute basis after dextran injection. The relative fluorescence intensity in the parenchyma was defined as ΔF = (F − F0)/F0, where F and F0 are fluorescence intensity at any given time point and the initial time point, respectively (63, 64).

### Data availability.

The RNA-Seq data have been deposited in Gene Expression Omnibus DataSets (https://www.ncbi.nlm.nih.gov/gds; accession no. GSE225065).

### Statistics.

Data were analyzed using a 2-tailed unpaired Student *t* test by comparing the 2 groups (control versus. *smLrp1^–/–^*) separately in the mice with *APOE3* or *APOE4* background. When comparing the association between fEPSP slope and fiber volley amplitude, repeated-measures 1-way ANOVA was used. *P* < 0.05 was considered significant. All statistical analyses were performed with GraphPad Prism 8.

### Study approval.

All animal procedures were approved by the Animal Study Committee at Mayo Clinic and in accordance with the regulations of the American Association for the Accreditation of Laboratory Animal Care.

## Author contributions

HO, GB, and TK designed research; HO, YY, WQ, CY, AK, FS, TMP, RBP, KK, NW, SCS, BR, IEM, TA, MLH, CCL, and YI performed research; HO, YR, PMS, YWA, BYSK, GB, and TK analyzed data; and HO and TK wrote the first draft of the manuscript.

## Supplementary Material

Supplemental data

## Version 1 04/10/2023

Electronic publication
