# Supplementary material for: LRP1 in vascular mural cells modulates cerebrovascular integrity and function in the presence of APOE4
Source: JCI Insight. 2023 Apr 10;8(7):e163822. doi: 10.1172/jci.insight.163822 (PMC10132158; doi:10.1172/jci.insight.163822)
Supplement: Supplemental data [file jciinsight-8-163822-s117.pdf]

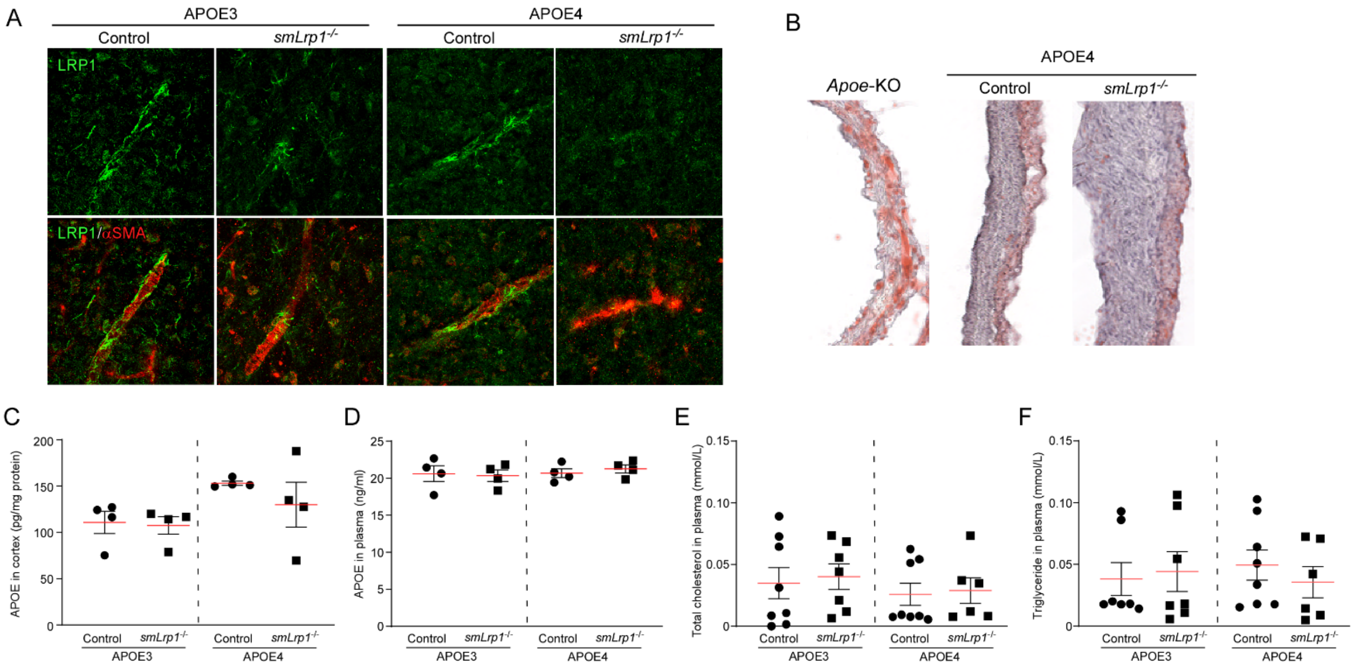

Supplemental Figure 1. Vascular mural cell specific LRP1 knockout mice with *APOE3* or *APOE4*. (A) Penetrating arteries in brain parenchyma from APOE3; control, APOE3; *smLrp1*$^{-/-}$, APOE4; control, and APOE4; *smLrp1*$^{-/-}$ mice were stained for LRP1 and a vascular mural cell marker α-smooth muscle actin (αSMA). (B) Hematoxylin–eosin and Oil Red O staining in aorta from *Apoe*-KO mice and the 13-16-month-old APOE4; control, and APOE4; *smLrp1*$^{-/-}$ mice. ApoE levels in the cortex (C) and plasma (D) from the 13-16-month-old male mice were measured by ELISA (N=4/group). Plasma concentration of total cholesterol (E) and triglyceride (F) from the 13-16-month-old male mice were measured were measured using Amplex™ Red Cholesterol Assay Kit (A12216) and Triglyceride Assay Kit (ab65336) (N=6-8/group), respectively. Bars represent mean ± SEM. Not significant by Student's t test between control and *smLrp1*$^{-/-}$ mice in each *APOE* genotype.

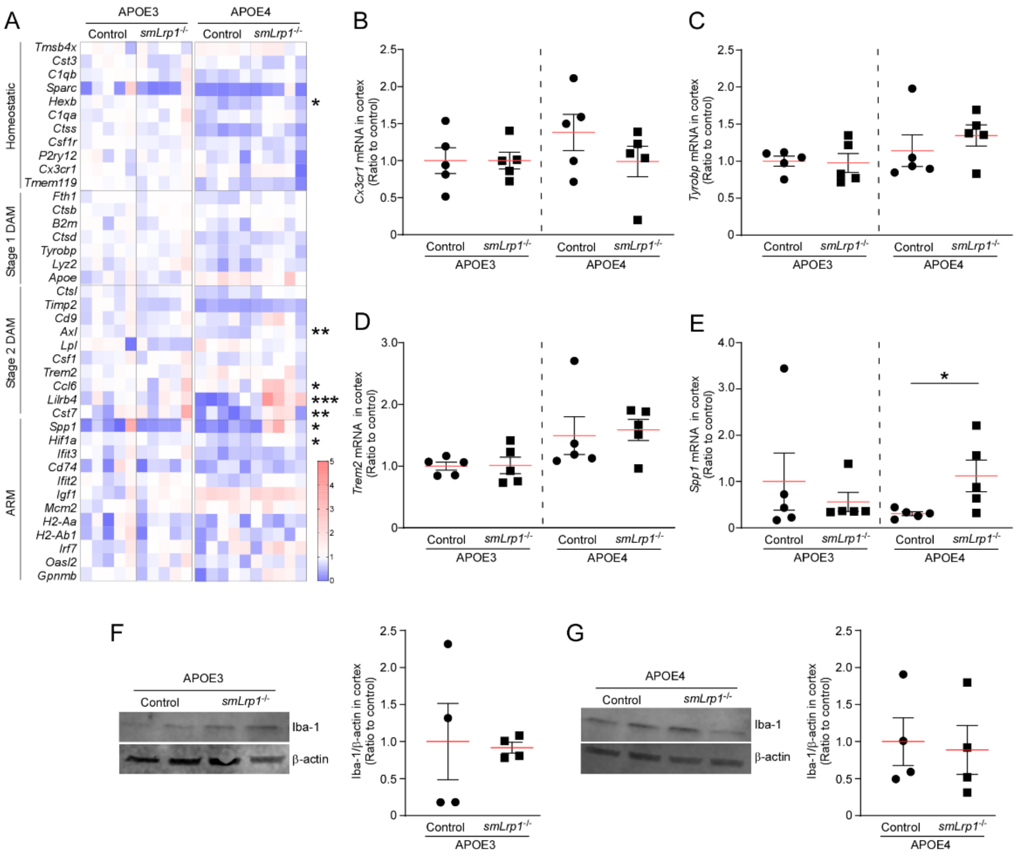

Supplemental Figure 2. Influences of LRP1 deletion in vascular mural cells on the microglia gene expressions. (A) Heatmap of selected major microglial gene expression through the RNA-seq data is shown. *p<0.05; **p<0.01; ***p<0.001, APOE4; control versus APOE4; *smLrp1*$^{-/-}$ mice. (B-E) The mRNA expression of *Cx3cr1* (B), *Tyrobp* (C), *Trem2* (D), and *Spp1* (E) were measured by RT-qPCR in the cortical samples from 13-16-month-old male APOE3; control, APOE3; *smLrp1*$^{-/-}$, APOE4; control, and APOE4; *smLrp1*$^{-/-}$ mice (N=5/group). Each mRNA expression was normalized to *Hprt* mRNA expression and shown as a ratio to that of APOE3; control mice. (F-G) Protein levels of Iba-1 in the in the cortical samples from 13-16-month-old male APOE3; control, APOE3; *smLrp1*$^{-/-}$, APOE4; control, and APOE4; *smLrp1*$^{-/-}$ mice were quantified by Western blotting (N=4/group). Bars represent mean ± SEM. *p<0.05 by Student's t test between control and *smLrp1*$^{-/-}$ mice in each *APOE* genotype.

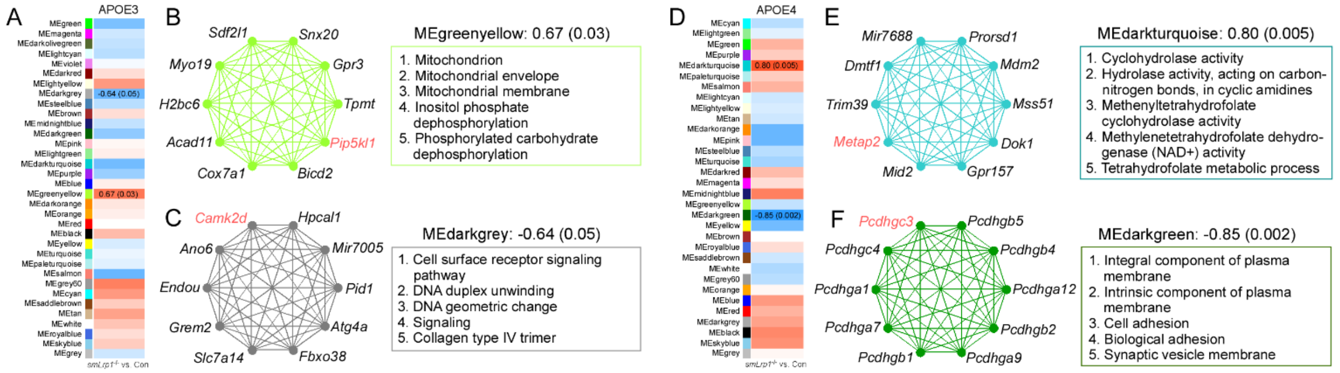

Supplemental Figure 3. Influences of LRP1 deletion in vascular mural cells on the gene co-expression networks of the brain transcriptomes. The correlation between module eigengenes (MEs) and LRP1 deficiency in vascular mural cells through WGCNA among the RNA-seq data from mice with *APOE3* (A) and *APOE4* (D) background is shown. The values in the heatmap are Pearson's correlation coefficients with p values. Modules with positive values (red) indicate positive correlation of MEs with the LRP1 deficiency; modules with negative values (blue) indicate negative correlation of MEs with these traits. Network plots of the top 10 genes with the highest intramodular connectivity in each module correlated with the LRP1 deficiency in the mice with *APOE3* (B; MEgreenyellow and C; MEdarkgrey), and the top 5 GO terms are shown. Network plots of the top 10 genes with the highest intramodular connectivity in each module correlated with the LRP1 deficiency in the mice with *APOE4* (E; MEdarkturquoise and F; MEdarkgreen), and the top 5 GO terms are shown. The top hub gene in each module is specified in red.

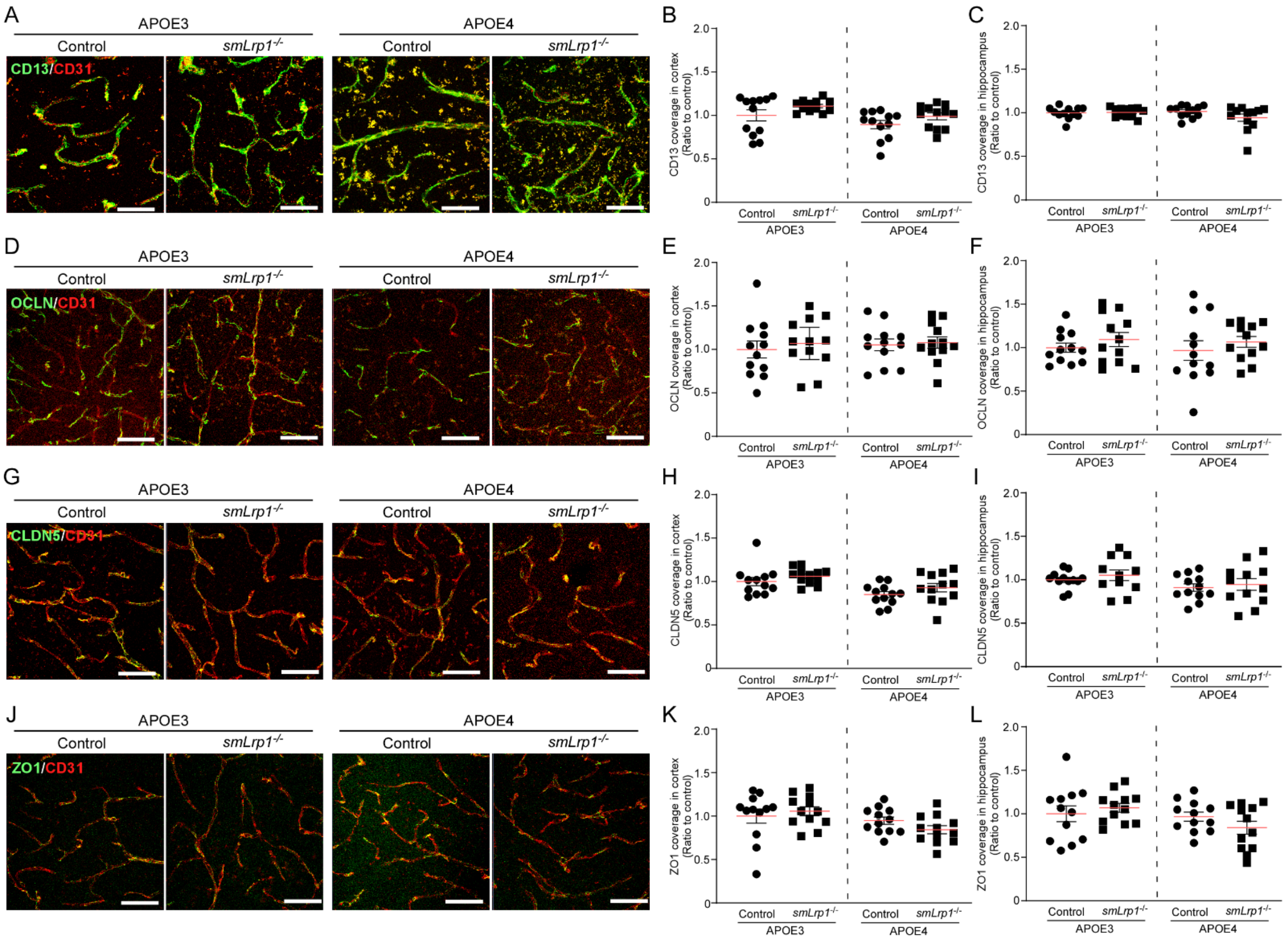

Supplemental Figure 4. LRP1 deletion in vascular mural cells does not influence the coverage of brain capillaries by pericytes and tight junction proteins. CD13 (A), OCLN (D), CLDN5 (G), and ZO1 (J) were stained with CD31 in frozen brain sections from 13-16-month-old male APOE3; control, APOE3; *smLrp1*$^{-/-}$, APOE4; control, and APOE4; *smLrp1*$^{-/-}$ mice. Scale bars; 50 µm. The % of coverage against CD31-positive endothelial by CD13 (B, C), OCLN (E, F), CLDN5 (H, I), and ZO1 (K, L) in the cortical and hippocampal sections was quantified by ImageJ software (11-12 regions from 4 mice/group) and shown as a ratio to that of APOE3; control mice. Bars represent mean ± SEM. Not significant by Student's t test between control and *smLrp1*$^{-/-}$ mice in each *APOE* genotype.

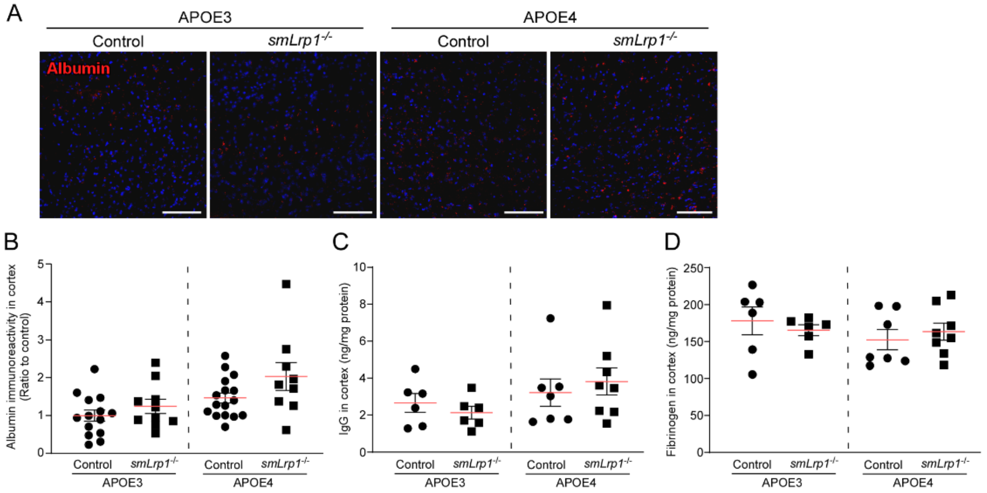

Supplemental Figure 5. LRP1 deletion in vascular mural cells does not influence leakages of large molecules from the blood flow into the brain. (A) Frozen brain sections from 13-16-month-old male APOE3; control, APOE3; *smLrp1*$^{-/-}$, APOE4; control, and APOE4; *smLrp1*$^{-/-}$ mice were stained for albumin. Scale bars; 100 µm. (B) Total fluorescence intensity of albumin in the cortical sections was quantified by ImageJ software (9-16 regions from 4 mice/group) and shown as a ratio to that of APOE3; control. (C, D) The levels of IgG (C) and fibrinogen (D) in the cortex from 13-16-month-old male APOE3; control, APOE3; *smLrp1*$^{-/-}$, APOE4; control, and APOE4; *smLrp1*$^{-/-}$ mice were determined by ELISA (N=6-8/group) and shown as a ratio to that of APOE3; control. Bars represent mean ± SEM. Not significant by Student's t test between control and *smLrp1*$^{-/-}$ mice in each *APOE* genotype.
